# RNA helicase MTR4 drives tumorigenesis of nasopharyngeal carcinoma by regulating the expression of key cell cycle genes

**DOI:** 10.1093/procel/pwac003

**Published:** 2022-10-14

**Authors:** Lili Yu, Lei Jiang, Meng Wu, Wenlong Dou, Kaiyuan Ji, Jianlong Zhou, Jinchul Kim, Yang Xu

**Affiliations:** Department of Cardiology, Cardiovascular Key Lab of Zhejiang Province, The Second Affiliated Hospital, School of Medicine, Zhejiang University, Hangzhou 310009, China; Department of Medical Oncology, Key Laboratory of Cancer Prevention and Intervention, Ministry of Education, The Second Affiliated Hospital, School of Medicine, Zhejiang University, Hangzhou 310009, China; Cancer Center, Zhejiang University, Hangzhou 310058, China; School of Basic Medical Sciences, Southern Medical University, Guangzhou 510515, China; Department of Cardiology, Cardiovascular Key Lab of Zhejiang Province, The Second Affiliated Hospital, School of Medicine, Zhejiang University, Hangzhou 310009, China; School of Basic Medical Sciences, Southern Medical University, Guangzhou 510515, China; School of Basic Medical Sciences, Southern Medical University, Guangzhou 510515, China; School of Basic Medical Sciences, Southern Medical University, Guangzhou 510515, China; Ageing Research Center, Korea Research Institute of Bioscience and Biotechnology, Deajeon 34141, Korea; Department of Cardiology, Cardiovascular Key Lab of Zhejiang Province, The Second Affiliated Hospital, School of Medicine, Zhejiang University, Hangzhou 310009, China

**Dear Editor,**

The pathogenesis of nasopharyngeal carcinoma (NPC) is closely associated with the infection of Epstein Barr virus (EBV) (Hau et al., 2020). However, the mechanisms underlying NPC tumorigenesis remain unclear, leading to a lack of therapeutic targets to effectively treat NPC. RNA helicase MTR4, which is a component of the virus-induced cofactor complex linking viral RNA to exosome for degradation, is overexpressed in NPCs of patients. The expression levels of MTR4 are inversely correlated with the prognosis of NPC patients. The silence of MTR4 in NPC cells suppresses the proliferation and colony-forming ability of NPC cells *in vitro,* and effectively inhibits the growth of NPCs formed by NPC cell lines and patients’ samples *in vivo*, indicating the importance of MTR4 in NPC tumorigenesis. MTR4 promotes NPC development by promoting the cell cycle progression of NPC cells. In this context, MTR4 maintains the expression levels of its target mRNAs, some of which are important cell cycle genes such as *CDK2*. The restoration of CDK2 expression in NPCs after MTR4 knockdown (KD) partially rescues the proliferation of NPCs *in vitro* and tumorigenesis *in vivo*, indicating that MTR4 drives NPC tumorigenesis by maintaining the expression levels of key target cell cycle genes. Therefore, MTR4 is a promising therapeutic target to treat NPC. Our findings also suggest a common mechanism to functionally link viral infection to cancer development, in which EBV takes advantage of the host defense mechanisms to promote NPC.

RNA decay is an anti-viral host defense system that senses viral RNA and suppresses viral replication through viral RNA degradation by the RNA exosome, the RNA processing/degradation complex responsible for 3ʹ exonuclease degradation of RNA (Nayak et al., 2017). The core of RNA exosome complex is composed of cofactors such as TRAMP (Trf4/5-Airl/2-MTR4 polyadenylation) complex and Rrp44/Rrp6 to target RNA for degradation (Houseley et al., 2006). RNA helicase MTR4 in the complex has anti-viral activities by recognizing viral mRNAs and unwinding RNA structures to make easy access of viral RNAs to exosome, promoting viral RNA decay (Houseley et al., 2006; Molleston et al., 2016).

To examine the potential involvement of MTR4 in the tumorigenesis of NPCs, we examined the expression levels of MTR4 in NPCs. By immunohistochemistry analysis of chips of NPC patient samples, we found that the MTR4 protein levels were inversely correlated with the prognosis of the NPC patients (Fig. 1A and 1B). In addition, 27 NPC transcriptome datasets were retrieved and constructed from the Gene Expression Omnibus (GEO) database. The expression levels of MTR4 were apparently increased in NPC samples when compared to non-tumor control tissues (Figs. 1C and S1A). To examine the importance of MTR4 in NPC tumorigenesis, the expression of MTR4 in NPC cells was silenced (Figs. S1B and S2). The silence of MTR4 expression in multiple NPC lines suppressed their proliferation and colony-forming ability, suggesting that MTR4 is important for NPC tumorigenesis (Fig. S2). To further examine the oncogenic functions of MTR4 in NPC development *in vivo*, MTR4 KD and control NPC cells were subcutaneously injected into immunodeficient mice to form NPC tumors, indicating that MTR4 is required for NPC tumor growth *in vivo* (Figs. 1D, 1E and S1C).

**Figure 1.**
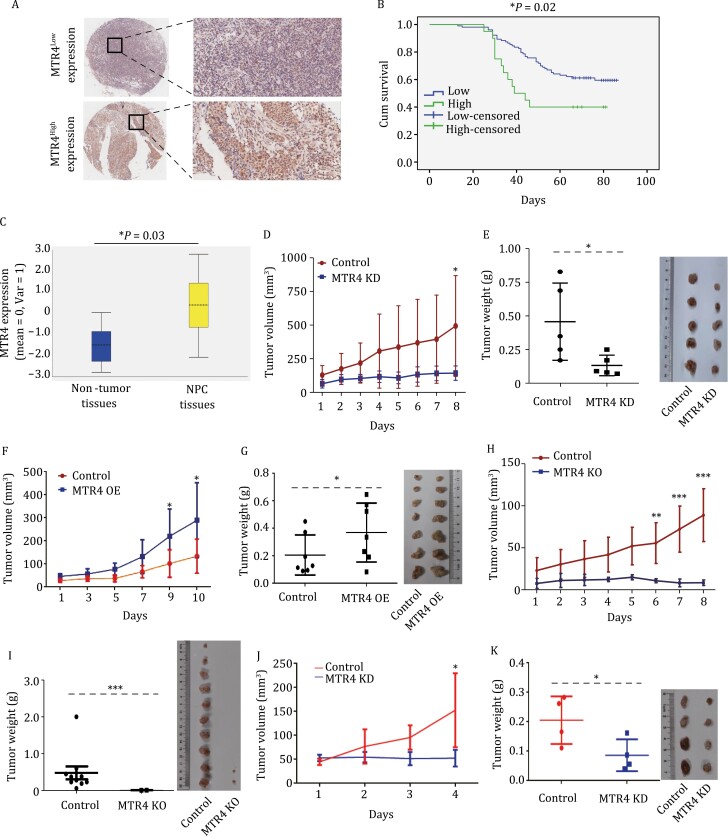
MTR4 is overexpressed in NPCs and required for NPC tumorigenesis. (A) Chips of NPC samples were stained with anti-MTR4 antibody and the intensity of the staining scanned and scored. Representative immunohistochemistry (IHC) images are shown. (B) The *MTR4* expression levels were inversely correlated with the postoperative recurrence-free survival (RFS) of NPC patients. The differences in survival rates were assessed with the log-rank test (Mantel Cox). The survival probability of the patients with high *MTR4* mRNA levels is significantly lower than those with lower *MTR4. n* = 20 for MTR4^high^ tumors. *n* = 103 for MTR4^low^ tumors. *P* value is indicated. (C) Box plot showing the relative mRNA levels of *MTR4* in NPC tissues and non-tumor tissues from GSE13597 dataset. The significance was assessed by two-tailed, unpaired *t*-test and *P* value is indicated. Centre is median, bounds of the box spans the interquartile range (from 25% to 75% percentile), and whiskers visualize minima and maxima. (D) The volumes and (E) the weight of the tumors formed by NPC cells expressing non-specific scramble shRNA (SC) or MTR4 specific shRNA cells in NSG mice were measured for each group. *n =* 6. Values represent the mean ± s.d. Repeated measures two-way ANOVA, followed by Bonferroni post-tests. ***P < 0.001. (F) The volumes and (G) the weight of tumors formed by control NPC cells or MTR4 overexpressed (OE) cells in NSG mice were measured for each group. *n =* 7. Values represent the mean ± s.d. Repeated measures two-way ANOVA, followed by Bonferroni post-tests. At the end of the treatment, the weight of all tumors in each group was compared. *n* = 7 for each group. Values represent the mean ± s.d. Mann-Whitney test. *P < 0.05. (H) The volumes and (I) the weight of tumors formed by MTR4^+/+^ or MTR4^+/−^ cells in nude mice were measured for each group. *n* = 10. Only 2 of 10 implanted MTR4^+/−^ NPC cells developed detectable tumors. Values represent the mean ± s.d. Repeated measures two-way ANOVA, followed by Bonferroni post-tests. At the end of the treatment, the weight of all tumors in each group was compared. *n* = 10. Values represent the mean ± s.d. Mann-Whitney test. ***P < 0.001. (J) The volumes and (K) the weight of NPC PDX tumors after MTR4 KD and scramble control. *n* = 4. Values represent the mean ± s.d. Repeated measures two-way ANOVA, followed by Bonferroni post-tests. *P < 0.05.

To further confirm the importance of MTR4 in NPC tumorigenesis, CRISPR/CAS 9 system was employed to knockout the MTR4 gene in NPCs. We failed to obtain homozygous MTR4 knockout cells, suggesting that MTR4 might be required for the survival of NPC cells (Fig. S1D). The knockout of one allele of the MTR4 gene in NPCs was sufficient to inhibit NPC tumorigenesis *in vitro* and *in vivo* (Figs. 1H, 1I and S1D). In addition, the overexpression of MTR4 in multiple NPCs promoted tumor growth (Figs. 1F, 1G, S1E and S2). To further confirm the importance of MTR4 in NPC development, we implanted NPC tissues derived from cancer patients into immunodeficient NSG mice to develop the NPC PDX model. To reduce the levels of MTR4 in NPC tumors of the PDX models, the NPC tumors were intratumorally injected with lentivirus expressing MTR4 shRNA. The mRNA levels of MTR4 were efficiently reduced in NPCs of PDX mice, leading to a significant inhibition of NPC growth in PDX mice (Figs. 1J, 1K and S1F). Together, these results demonstrate that MTR4 is important for NPC tumorigenesis *in vitro* and *in vivo*.

To reveal the mechanisms underlying the MTR4-dependent tumorigenesis of NPCs, the global gene expression was profiled after the inducible knockdown of the MTR4 gene in NPCs (Figs. S1G and S3A). Our results showed that the differentially expressed genes (fold change > 2.0) were significantly enriched in lipid metabolism and cell cycle pathways (Fig. S3B). Among the enriched pathways, cell cycle pathways stood out because they are included in the top 2 pathways to which MTR4-binding transcripts were annotated. When we clustered the expression of genes associated with cell cycle into a group, there was apparent difference between the control and MTR4 KD cells (Fig. 2A). Gene Set Enrichment Analysis (GSEA) further revealed that the cell cycle pathway was significantly deregulated in the MTR4 KD NPC cells (Fig. S3C). To reveal the clinical relevance of the expression levels of MTR4 and cell cycle machinery, we analyzed the correlation between the expression levels of MTR4 and the cell cycle-related genes in NPC transcriptome dataset obtained from the GEO database. Consistent with data of RNA-seq analysis, the expression levels of MTR4 in NPC tissues were correlated to the expression levels of genes with key roles in cell cycle (Fig. 2B–G).

**Figure 2.**
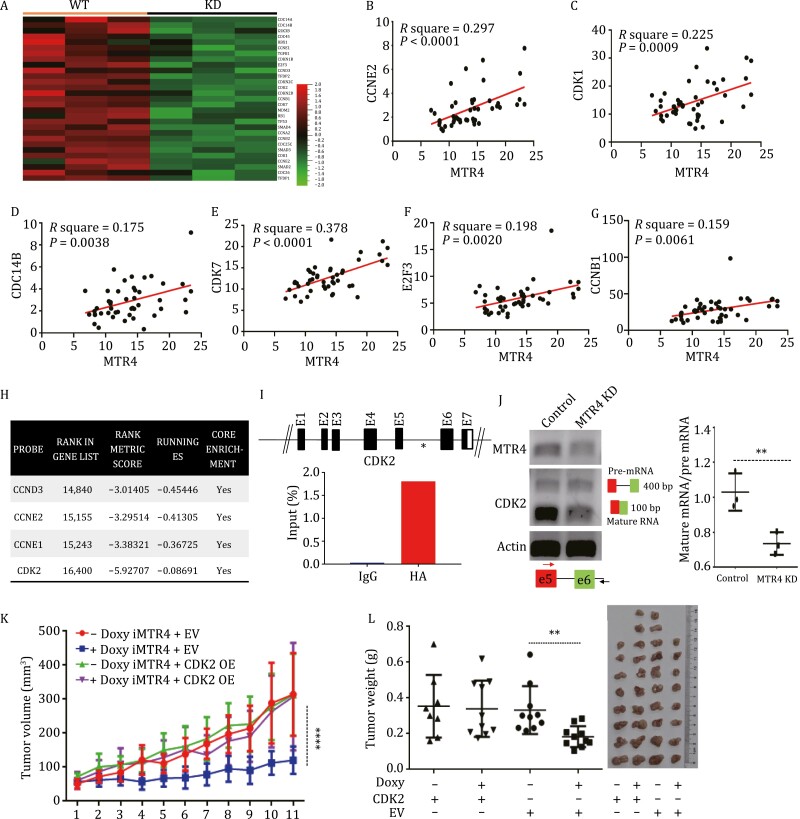
MTR4 promotes cellular proliferation of NPCs. (A) Heat map of the mRNA expression profile in cell cycle pathway genes in MTR4 inducible knockdown (iMTR4) cells before and after Doxy treatment. (B–G) The expression levels of MTR4 in NPC tissues were correlated to the expression levels of genes with key roles in cell cycle from the GSE13597 dataset. (H) pre-mRNAs bound by MTR4 were identified by RIP-seq analysis. (I) RIP analysis to confirm the binding of MTR4 to the CDK2 pre-mRNA. A schematic of CDK2 gene displaying a potential binding motif of MTR4 indicated by an asterisk. *n* = 2. (J) The levels of pre-mRNA and mRNA of CDK2 in MTR4 KD cells versus control cells were detected by semi-quantitative PCR and normalized by actin levels*. n* = 3. Values represent means ± s.d. *P < 0.05 and **P < 0.01. (K, L) The volumes (K) and the weight (L) of tumors formed by indicated cells in NSG mice were measured (*n* = 8 or *n* = 10 for each group). Two-way ANOVA, followed by Bonferroni post-tests. *P < 0.05 and **P < 0.01.

Consistent with the findings that MTR4 knockdown reduces the expression of cell cycle genes, the inducible knockdown of MTR4 decreased cell cycle progression of NPC cells (Fig. S3D and S3E). By comparing the MTR4-bound genes (Yu et al., 2020) with DEGs after MTR4 KD in NPCs, we predicted that MTR4 might directly regulate the expression of the key cell cycle genes by binding directly to these genes (Fig. 2H). We further validate the binding of MTR4 to the mRNAs of these cell cycle genes such as CDK2 gene with the predicted binding site and RNA immunoprecipitation (RIP) analysis (Figs. 2I and S3F). The residue 999 mutant of MTR4 (MT999) within the RNA binding motif of MTR4 inactivates the binding of MTR4 to its target RNA (Fig. S3F). The reduction in the binding of MTR4 R999A mutant to the CDK2 RNA further supports the specific interaction between MTR4 and the target RNAs. Since MTR4 is known to play key roles in regulating alternative splicing of its target pre-mRNA (Yu et al., 2020), the reduction of its target mRNA could be due to the defective alterative splicing of pre-mRNA. While MTR4 KD did not affect the levels of CDK2 pre-mRNA, it reduced the levels of CDK2 mRNA, suggesting that MTR4 maintains CDK2 mRNA levels via alternative splicing (Fig. 2J).

To investigate the importance of MTR4-dependent cell cycle gene expression in NPC tumorigenesis, the CDK2 gene was overexpressed in NPCs with or without MTR4 KD. The overexpression of CDK2 restored the expression levels of CDK2 in NPC cells after MTR4 knockdown (Fig. S4A). This rescued the defective proliferation, colony formation, and tumorigenesis of NPC cells after MTR4 knockdown (Figs. 2K, 2L, S4B and S4C). These data demonstrate that MTR4 drives the tumorigenesis of NPCs by maintaining the expression levels of cell cycle genes such as *CDK2*.

In search for new therapeutic targets of NPCs, we are focusing on MTR4, a member of SKI2-like RNA helicase family, expression of which is induced by EBV infection. We discovered that the silencing of MTR4 in NPC cell lines can significantly reduce their cell cycle progression and thus its tumorigenic potential *in vitro* and *in vivo*. Our findings also show that MTR4 could regulate lipid metabolism, suggesting another possible mechanism for MTR4 to promote NPC tumorigenesis. More importantly, the silencing of MTR4 in the tumors of NPC PDX models also dramatically suppresses the tumor growth, indicating that MTR4 is a common driver of NPC tumorigenesis despite the heterogeneity of NPC. Therefore, MTR4 represents a promising target for NPC. These findings also reveal a novel mechanism for EBV to take advantage of the host defense mechanism to promote NPC tumorigenesis.

It has been conservatively estimated that viral infections are responsible for at least 12% of the global cancer cases (Parkin, 2006). In supporting of this notion, whole genome sequencing data from 2,658 cancers across 38 multiple types of tumor indicated that 16% of them is virus-associated (Zapatka et al., 2020). Intriguingly, based on our published work and data present here, HCC and NPC, both viral-etiological cancers with HBV and EBV infection respectively, depend on MTR4 for tumorigenesis (Yu et al., 2020). Therefore, we speculate that the RNA helicases such as MTR4 could serve as a functional link between deregulated host defense mechanism and tumorigenesis during chronic viral infection. If so, MTR4 can be developed as a therapeutic target and diagnosis biomarker in virus-mediated HCC and NPC.

## Supplementary Material

pwac003_suppl_Supplementary_Material

## Footnotes

This research was supported by the National Natural Science Foundation of China (Grant Nos. 81902506, 81930084).

The authors have no relevant financial or non-financial interests to disclose.

All procedures followed were in accordance with the ethical standards of the responsible committee on human experimentation (institutional and national) and with the Helsinki Declaration of 1975, as revised in 2000 (5). Informed consent was obtained for NPC tissues in this study. The use and care of animals complied with the guidelines of the Institutional Animal Care and Use Committees. All data included in this published article are available from the corresponding authors upon request.

L. Yu and Y. Xu conceptualized, designed, and supervised the research; L. Yu, L. Jiang, M. Wu, W. Dou, K. Ji, J. Zhou, and J. Kim conducted experiments; L. Yu, L. Jiang and Y. Xu analyzed the data; L. Yu, J. Kim, and Y. Xu wrote the manuscript. All authors revised and approved the manuscript.
