# Supplementary material for: RNA helicase MTR4 drives tumorigenesis of nasopharyngeal carcinoma by regulating the expression of key cell cycle genes
Source: Protein Cell. 2022 Oct 14;14(2):149–52. doi: 10.1093/procel/pwac003 (PMC10019564; doi:10.1093/procel/pwac003)
Supplement: pwac003_suppl_Supplementary_Material [file pwac003_suppl_supplementary_material.pdf]

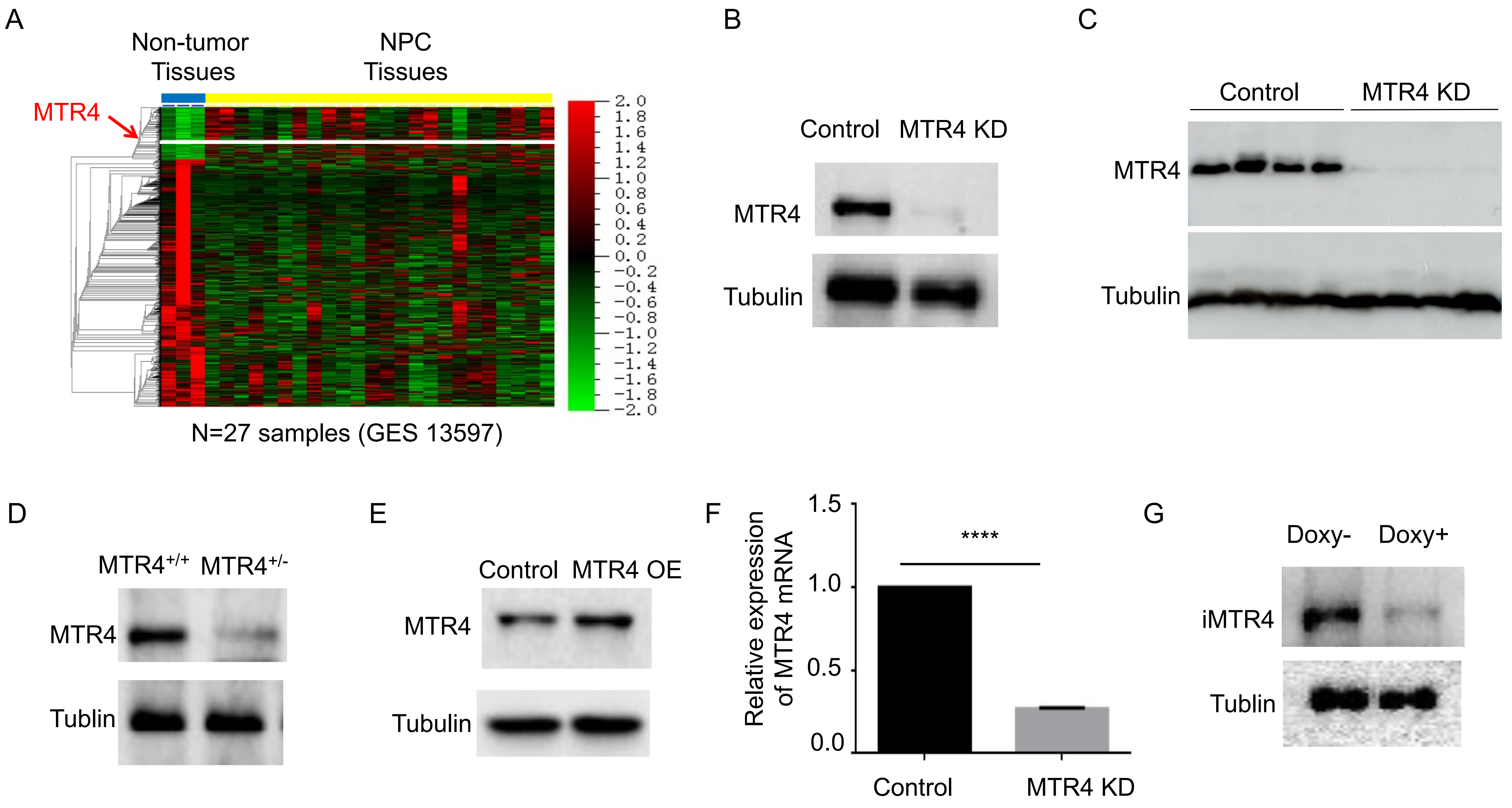

Supplementary Figure 1. MTR4 expression and NPC tumorigenesis. (A) Heat map of the global mRNA expression profile in non-tumor tissues and nasopharyngeal carcinoma (NPC) tissues from GSE13597 dataset. The levels of *MTR4* mRNA expression are indicated with a white line. (B,C) The knockdown of MTR4 expression in 5-8F NPC cells (B) and in NPC tumors (C) were confirmed by Western blotting. Tubulin was used as an internal control. Control cells were treated with non-specific scramble shRNA. (D) The heterozygous knockout of MTR4 gene in NPC cells was confirmed by Western blotting. Tubulin was used as an internal control. Control cells were MTR4 wild type NPC cells. (E) The overexpression of MTR4 gene in NPC cells (MTR4 OE cells) was confirmed by Western blotting. Tubulin was used as an internal control. (F) The intra-tumoral injection of lentiviral shRNA into NPC PDX tumors reduced the MTR4 mRNA levels. N=4. Values represent the mean ± s.d. (G) Inducible knockdown of MTR4 in NPC cells (iMTR4 cells) was confirmed by Western blotting after the treatment with 1μg/ml doxycycline (Doxy) for 3 days. Tubulin was used as an internal control.

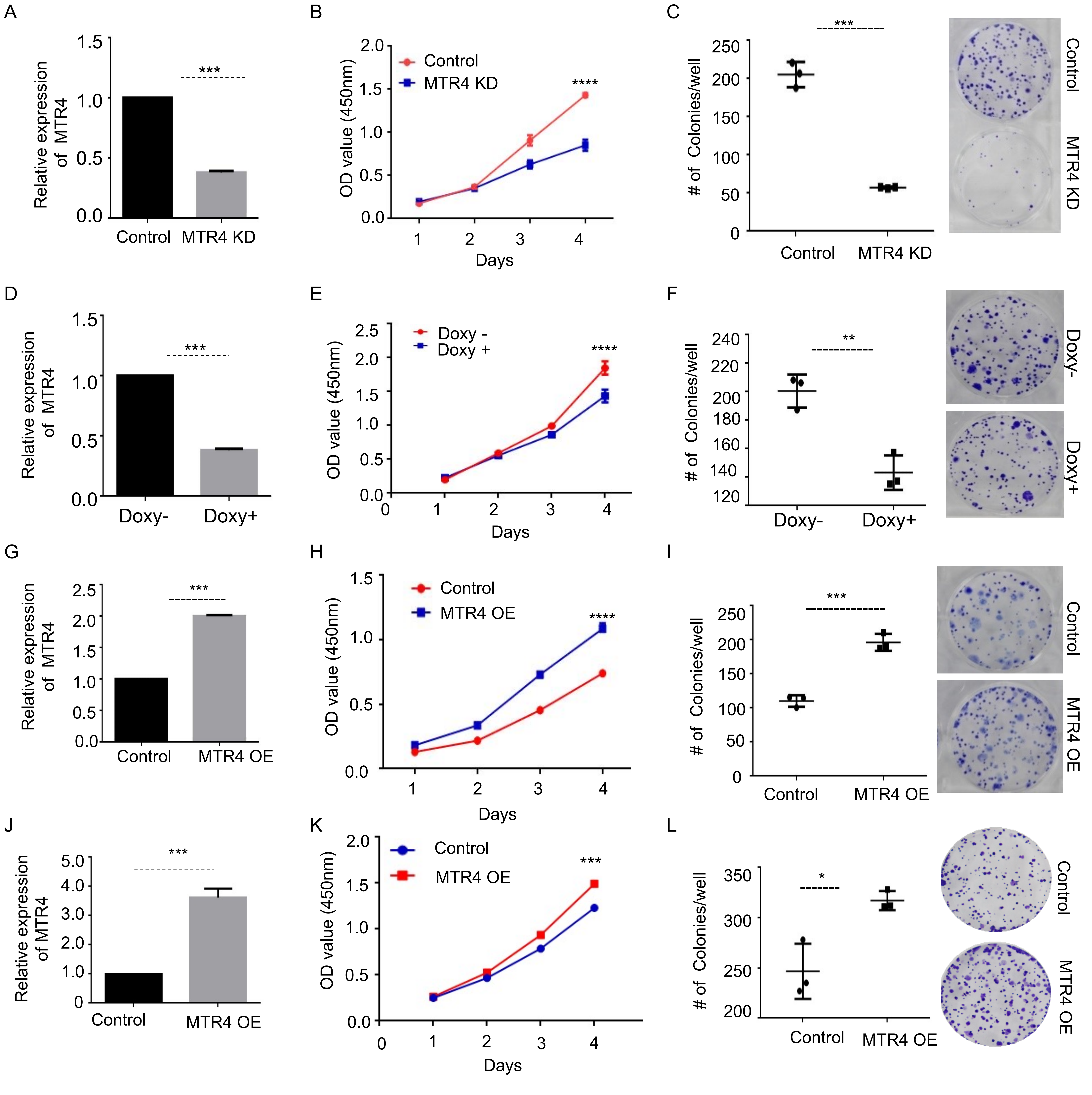
A
B
C
D
E
F
G
H
I
J
K
L
Relative expression of MTR4
OD value (450nm)
# of Colonies/well
Days
Control
MTR4 KD
Doxy -
Doxy +
Doxy-
Doxy+
MTR4 OE
***
****
**
*

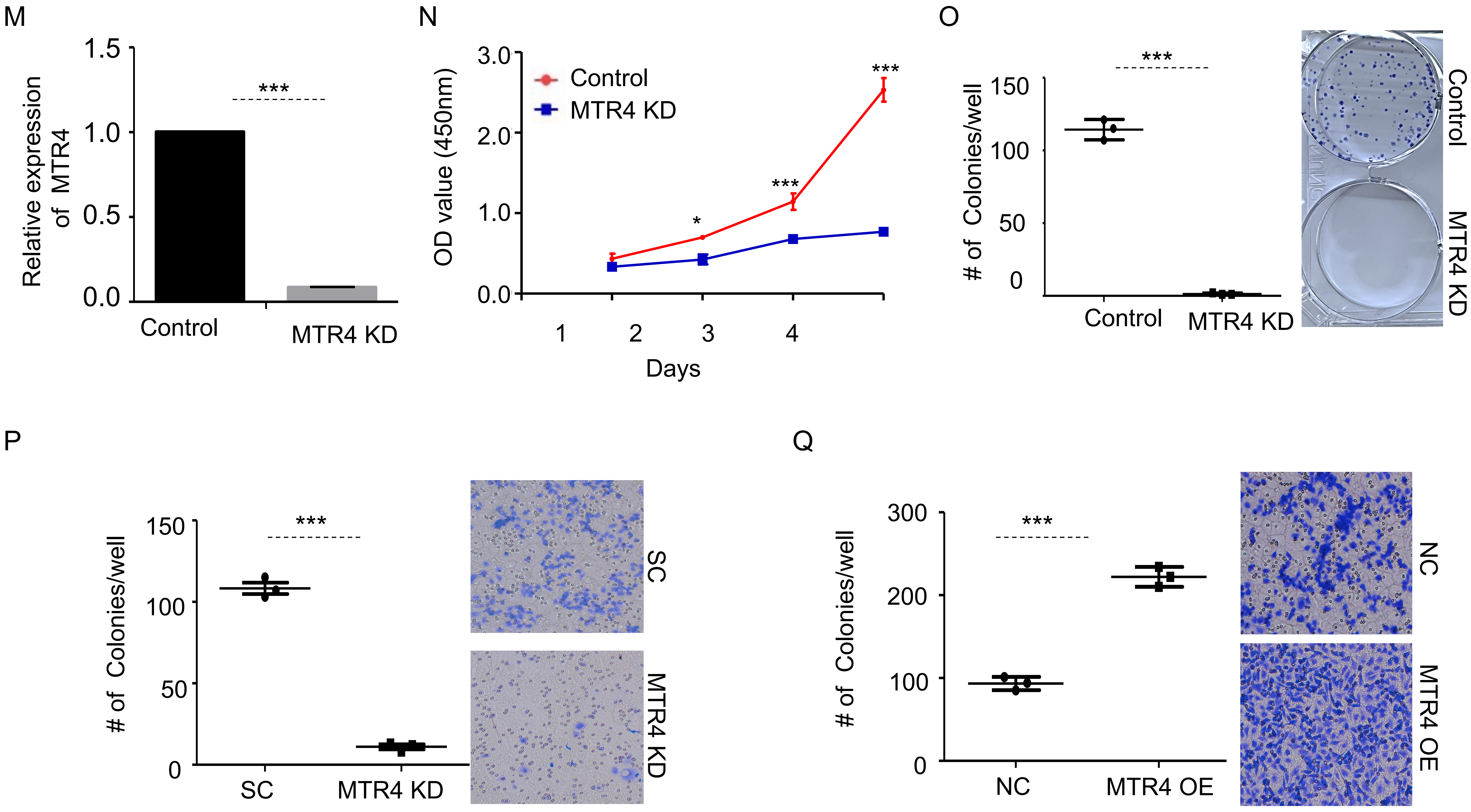

Supplementary Figure 2. MTR4 is required for NPC tumor growth *in vitro*. 5-8F NPC cells (A-C,G-I,), SNUE1 NPC cells (D-F, J-L) and HONE1 NPC cells (M-O) were infected with lenti-virus harboring doxycycline (Doxy)-inducible shRNA for MTR4 (iMTR4 KD) or lenti-virus harboring stable shRNA (MTR4 KD), respectively. Control cells were treated with virus harboring non-specific scramble shRNA. (A, D, G, J, M) The levels of *MTR4* mRNA after MTR4 KD or overexpression were confirmed by qPCR. Actin was used as an internal control. N=3. (B, E, H, K, N) Proliferation of indicated cells was analyzed with CCK8 assay. Data are presented as mean value ± s.d. N= 3. Difference between two groups was calculated by two-way ANOVA, followed by Bonferroni post-tests. ****p* <0.001, *****p* <0.0001. (C, F, I, L, O) Colony formation assay of indicated cells. Representative images showed colonies stained with crystal violet. The number of colonies in each well was counted. N = 3. Difference between two groups was calculated by two-tailed, unpaired t-test. Data are presented as mean value ± s.d. **p* < 0.05, ***p* <0.01, ****p* < 0.001. (P, Q) Migration assay of 5-8F NPC cells (P) and SNUE1 NPC cells (Q). Representative images showed colonies stained with crystal violet. The number of cells in each field was counted. Difference between two groups was calculated by two-tailed, unpaired t-test. Data are presented as mean value ± s.d. N= 3. ****p* <0.001.

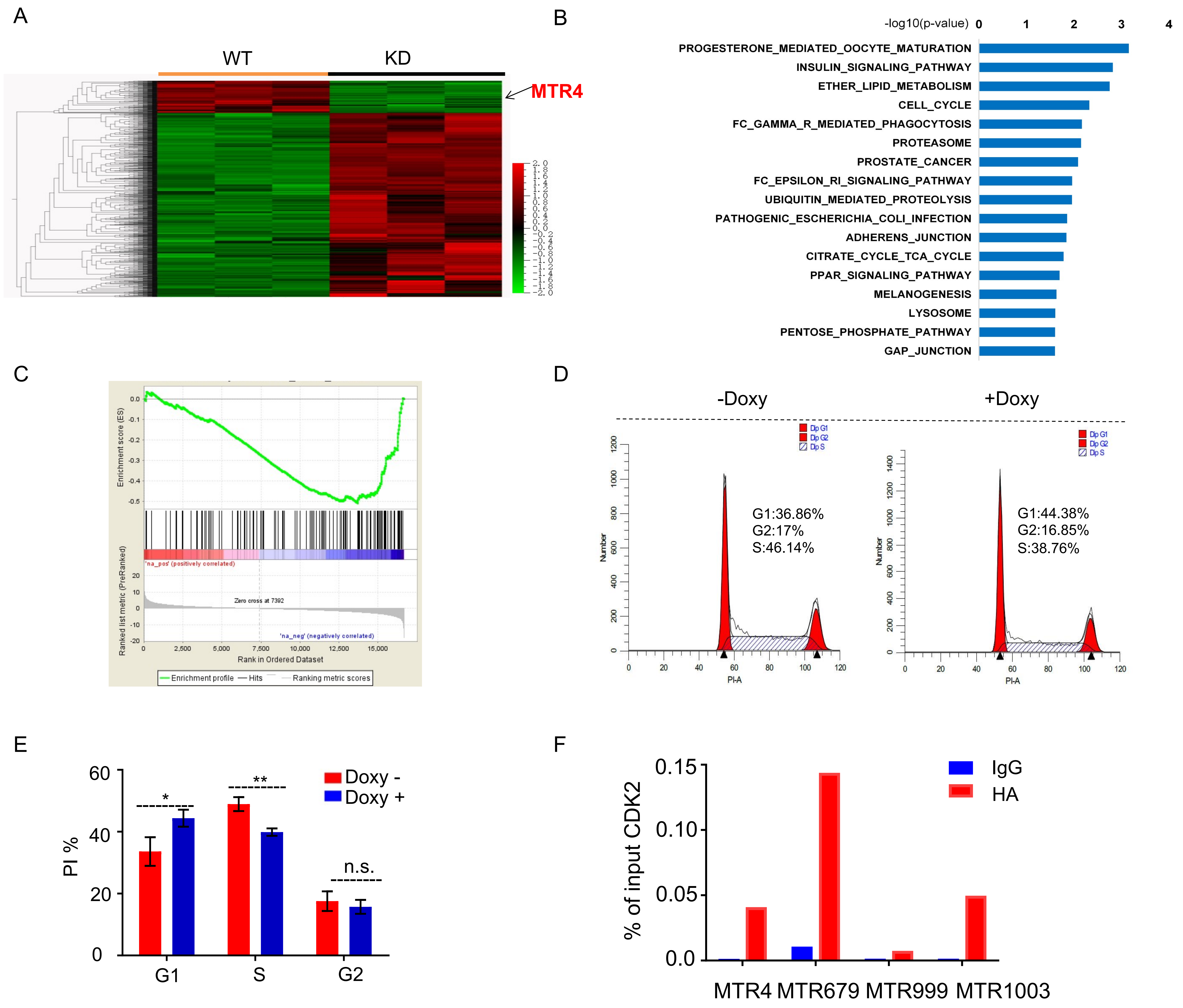

Supplementary Figure 3. MTR4 promotes the cell cycle progression of NPCs. (A) Heat map of the global mRNA expression profile in iMTR4 cells before and after Doxy treatment. The levels of *MTR4* mRNA are indicated with a white line. (B) Pathway enrichment analysis of differentially expressed genes (DEGs) in NPC cells before and after Doxy treatment. (C) Gene Set Enrichment Analysis (GSEA) further reveals that cell cycle pathway is significantly deregulated in the MTR4 KD NPC cells. Representative (D) and statistical (E) data of cell cycle distribution of NPCs before and after MTR4 KD is shown. N=3. Data are presented as mean value ± s.d. Unpaired t-test. **p* < 0.05, ***p* < 0.01, n.s. non-significant. (F) RIP analysis of HA-tagged MTR4 mutants (K679N , R999A, E1002A) in NPCs. N = 2.

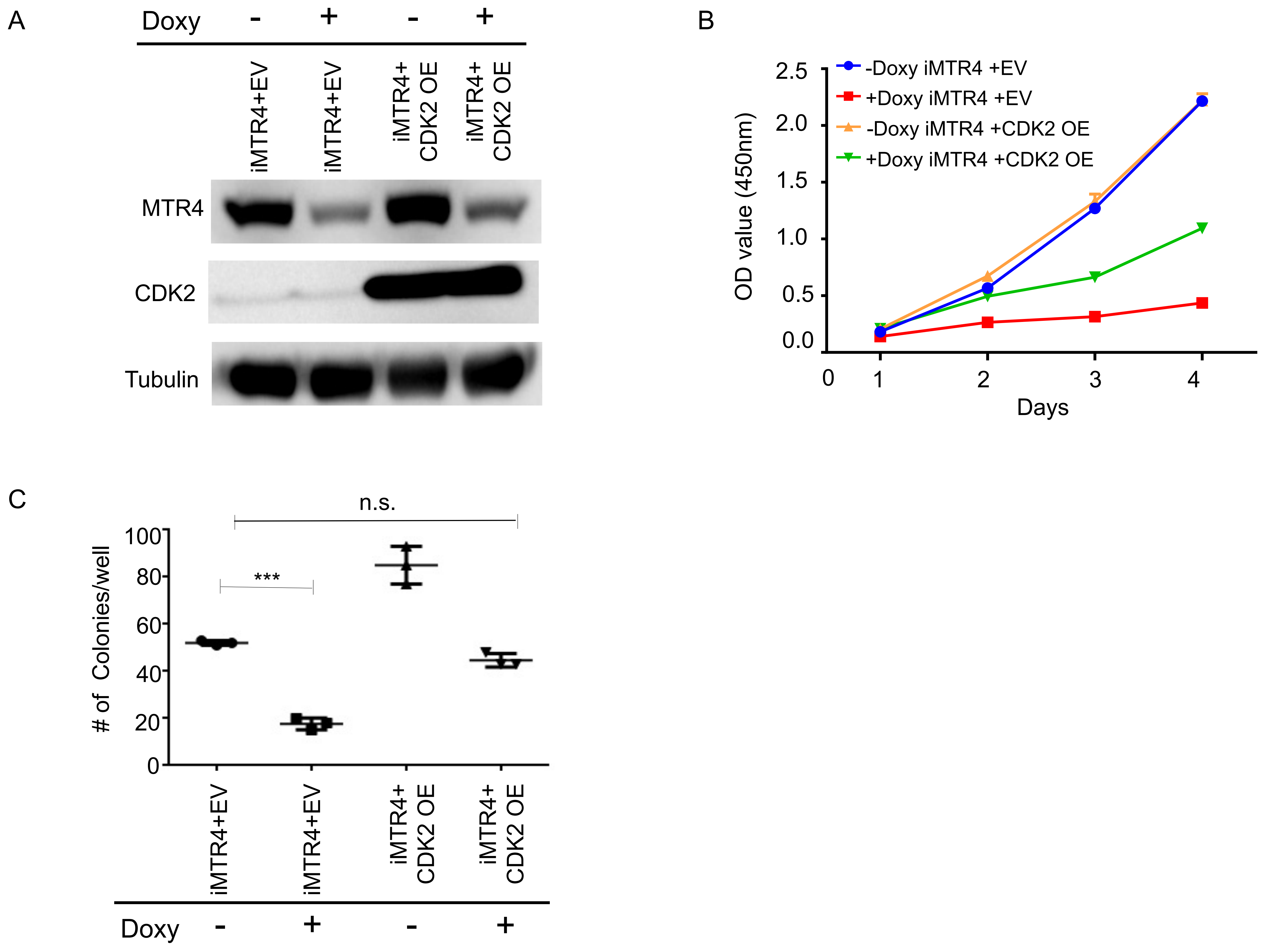

Supplementary Figure 4. Ectopic expression of CKD2 rescued the defective tumorigenesis of NPC cells after MTR4 KD. (A) Ectopic expression of CDK2 in control and MTR4 KD cells was analyzed for protein levels of MTR4 and CDK2 by Western blotting. (B) The proliferation of indicated cells was analyzed with CCK8 assay. N=3. Data are presented as mean value ± s.d. Statistics were calculated by two-way ANOVA with a Tukey's multiple comparison test. *P* values are indicated. (C) Colony formation of control cells and MTR4 KD cells transfected with either empty vector (EV) or plasmids expressing CDK2 (CDK2 OE). The number of colonies in each well was counted. N=3. Data are presented as mean value ± s.d. Significant difference between two groups was calculated by two-tailed, Unpaired two-tailed t-test. *** $p < 0.001$, n.s., non-significant.

Supplementary information

MATERIALS AND METHODS

Tissue Chip. Nasopharyngeal carcinoma **(**NPC) Tissue chip (HNasN132Su01, China) was purchased from Shanghai Outdo Biotech Company. The study was approved by the institution research ethics committee of Shanghai Outdo Biotech Company, and written informed consent was obtained from each subject. Chips of NPC samples (n = 133) were stained with anti-MTR4 antibody and the intensity of staining was scanned and according to the staining power or positive rate scored, the total score is their product. Representative immunohistochemistry (IHC) images are shown. Patients were divided into two groups based on the MTR4 levels: patients with high MTR4 expression in NPC (total score more than 6 points) and the rest of the patients. The postoperative survival rate was analyzed with the Kaplan–Meier method, and the difference in survival was assessed with the log-rank test. Univariate and multivariate analyses were based on the Cox proportional hazards regression model. All the statistical analyses were performed with IBM SPSS Statistics 20.0 (IBM, IL, USA). Two-sided p values were calculated, and $p < 0.05$ was considered a statistically significant result.

Cell lines, viral vectors, constructs, and transfection. 293 FT cell line was purchased from Thermo Fisher Scientific (Waltham, MA). Other cell lines were obtained from ATCC (American Type Culture Collection, USA) and maintained in our laboratory. 293 FT cell line was cultured at 37 °C with 5% CO_2 in DMEM and others were cultured in RPMI-1640 (Invitrogen, Carlsbad, CA) containing 10% fetal bovine serum (FBS) (Gibco,Australia). To establish cells with stably silenced gene, lenti-viruses expressing short hairpin RNA (shRNA) by the pLKO.1 puro vector were used (Addgene

#8453) according to Addgene's pLKO.1 protocol. MTR4 shRNA target sequence is GGAAGGATTTCCGATGGATTT. shRNA oligomers were designed, annealed, and inserted into PLKO.1 puro according to Addgene's pLKO.1 protocol. Lenti-viruses were produced by co-transfecting the pLKO.1 vectors, packaging plasmid psPAX2 (Addgene plasmid 12260, Addgene, Cambridge, MA) and envelope plasmid pMD2.G (Addgene plasmid 12259) into HEK293FT cells. For the doxycycline (Doxy)-inducible MTR4 knockdown, tet-pLKO-puro vector (Addgene #21915) and Fuw-M2rtTA (Addgene 20342) were used. Seventy-two hours postinfection, cell debris in the media was removed by centrifugation at 2000 rpm for 10 min and lenti-virus concentrated by Lenti-X concentrator (Clonetech, Mountain View, CA). One day before the viral infection, cells were plated into a six-well plate at a density of 1×10^5 cells/well and transduced in the presence of 5 μg/ml polybrene for 72 h. The cells were selected for puromycin resistance (2 μg/ml) for 7 days. To overexpress CDK2, the full length of CDK2 cDNA was inserted into pLenti-CMV GFP vector (Addgene 17448) linearized by Xba1 digestion.

Cell growth assay. For clonogenic assay, single-cell suspension was prepared by trypsinization and the indicated cell lines seeded onto 6-well plates with a density of 500 or 800 cells per well. After culturing for 2 weeks, colonies were stained with crystal violet and counted. Cell Counting Kit 8 (CCK8) assay was performed using 96-well plates. Briefly, cells were seeded at a density of 2-3 × 10^3 cells/well, and cultured for 24–96 h. CCK-8 solution was added to each well and incubated for 60 min, the plates were measured at 450 nm with a microplate reader and analysis.

Animal experiments . All animal work was approved by Institutional Animal Care and Use Committee (IACUC) of Southern Medical University. To analyze tumor growth in NODSCID mice, cells were harvested, resuspended in 0.15 ml DPBS mixed with Matrigel at an 2:1 ratio, and

injected subcutaneously into the left and right flank of NODSCID mice (2 × 10^6 cells each). To evaluate the impact of MTR4 depletion on tumorigenicity, mice bearing established tumors about 0.5 cm in diameter were randomized into two groups. Subsequently, Doxy (20 mg/kg body weight) was injected intraperitoneally (i.p.) every day. The control group was injected with the same volume of PBS. In addition, doxy-treated group was continuously provided with doxy containing drinking water (4 mg/L).

Subcapsular tumor transcription animal model. NPC 5-8F cells with or without MTR4 were infected with Ubi-MCS-firefly-Luciferase-IRES-Puromycin virus. To develop the subcapsular tumour transcription mouse model of NPC, male NOD/SCID mice aged 6 to 8 weeks were used. Mice were anesthetized, 1×10^6 5-8F cells with or without MTR4 were resuspended in 40 µl of DPBS mixed with Matrigel at an 1:1 ratio and subcapsular injection to establish the mouse model. Tumour growth was monitored by weekly bioluminescence imaging (BLI) for luciferase and mice were monitored daily for survival. Nod/ Scid mice bearing tumours were anesthetized using isoflurane and injected i.p. with 150 mg/kg D-luciferin (Molecular Probes, Carlsbad, CA, USA). BLI was measured with 18 filters (500–840 nm) in an IVIS Spectrum (PerkinElmer, Waltham, MA, USA) 10 min after injection. During image acquisition, mice were maintained on isoflurane via nose cone. Bioluminescence images were acquired using an IVIS Spectrum. BLI signal was reported as total flux (photons per second), which represents the average of ventral and dorsal flux. At the end-point of the study, the animals were injected with D-luciferase and sacrificed 10 min later. Organs were exposed and BLI was measured. After organs were excised, BLI images were taken again as described. Imaging analysis was performed using the Living Image software (Caliper Life Sciences, Waltham, MA, USA).

PDX Tissue Preparation and Subcutaneous PDX Implantation. NPC fresh tissues should be saved in cold RPMI-1640 media with 10% FBS after excised, and all PDX tissues preparation and inoculation should be performed in a biosafety cabinet using sterile tools and techniques. This cabinet should be decontaminated with ultraviolet radiation 30 minutes before use. Laboratory personnel should wear proper PPE to handle tumors. Place the solid tumor in a culture dish on a cold pack, and cut the tumor into pieces with scalpel, and then mince them with a pair of fine scissors and mixed with Matrigel at an 1:1 ratio. Load all the finely minced tumor into a 5-cc syringe without air bubbles, and then transfer tumors into a 1-cc syringe. Place the 14 gauge needle on the 1-cc syringe tip and injected subcutaneously into the left and right flank of NOD/SCID mice, 0.1 mL/point. The needle is reused for up to five mice within the same cage. Save some tumor fragment for histological analysis. To evaluate the impact of MTR4 depletion on tumorigenicity, NPC PDX (P3) model mice bearing established tumors about 0.5 cm in diameter were randomized into two groups. And treated with sh-MTR4 or scrambled virus (virus titer 1 to 3 E + 8 TU/mL, 50 µl /point) intro-tumor injection.

Western blotting. Samples were separated on 8–15% SDS PAGE and transferred to nitrocellulose membranes. The blots were incubated in blocking buffer (5% skim milk in PBS with 0.05% Tween 20) with primary antibodies. After washing three times with blocking buffer, the blots were probed with a horseradish peroxidase-conjugated secondary antibody and developed with Supersignal West Pico or Dura (Thermo Fisher Scientific). The following antibodies were used: rabbit polyclonal anti-MTR4 antibody (ab70551; Abcam), rabbit polyclonal anti-CDK2 antibody (10122-1-AP; Proteintech), mouse monoclonal anti-α-tubulin antibody (T5168; Sigma-Aldrich), anti-rabbit IgG, HRP-linked antibody (7074S; Cell Signaling), anti-mouse IgG antibody, HRP-linked

antibody (7076S; Cell Signaling). The intensity of protein bands was quantified using Image Lab (BioRad). All uncropped and unprocessed scans of western blots are provided as Supplementary. in the Supplementary Information.

RNA extraction and Quantitative PCR analysis. RNeasy Mini Kit (Invitrogen) was used to extract total RNA from cells according to the manufacturer's instructions. Cells were collected directly into RLT lysis buffer and homogenized using QIAshredder (Qiagen) according to manufacturer's instructions. RNA was purified with RNeasy Mini kitcolumns, and genomic DNA removed with RNase-free DNase Kit (Qiagen). TRIzol reagent (Thermo Scientific) was used for RNA extraction from human patient tissues. 0.5 mg of liver tissues was homogenized in 1 ml TRIzol reagent with electric homogenizer and mixed with 0.2 ml chloroform vigorously (Sigma-Aldrich). After incubating at room temperature for 3 min, the mixture was centrifuged at 10,000 × g for 15 min. RNA fraction was mixed with the same volume of 100% ethanol and purified with RNeasy column. cDNA synthesis from total RNA was performed using High Capacity cDNA Reverse Transcriptase Kit (Applied Biosystems) following the manufacturer's instructions. Real-time qPCR was performed with an ABI Prism 7000 (Applied Biosystems) using FastStart Universal SYBR Green Master (Takara). PCR conditions were following: 2 min at 95 °C, 40 cycles of 5 s at 95 °C, and 34 s at 60 °C. The average Ct value for each gene was determined from triplicate reactions and normalized with mRNA levels of β-actin or 18 s. Sequence of all primers used in qPCR analysis was provided in Supplementary.

RNA immunoprecipitation. RNA immunoprecipitation (RIP) was carried out with Magna RIP RNA-Binding Protein immunoprecipitation kit (Millipore; USA) according to the manufacturer's instruction. Cells overexpressing MTR4-HA were lysed by RIP lysis buffer containing protease

inhibitor cocktail (Thermo Fisher, USA) and RNase inhibitor (Invitrogen, USA). To obtain the plasmid expressing MTR4-HA, the full length of MTR4 cDNA was amplified with primers tagged with HA sequence (TACCCATACGATGTTCCAGATTACGCT) and inserted into pcDNA3 vector that was linearized with Kpnl/Notl digestion. Nuclear pellets were collected and lysed by sonication. Nuclear lysateswere incubated with anti-HA antibody or control IgG overnight at 4 °C, followed by incubation with protein A/G Dynabeads (Thermo Fisher, USA) for 2 h. After vigorous washing, pellets were resuspended in TRIzol (Invitrogen, USA). The immunoprecipitated RNAs were purified by RNAeasy Mini Kit (Invitrogen), reversely transcribed.

Immunohistochemistry (IHC) analysis of tumor samples. Tumor samples were fixed with 4% paraformaldehyde in 0.1 M phosphate buffer for 24 h, embedded in paraffin, sliced into 4-μm sections and mounted onto glass slides. After dewaxing, the slides were treated with 3% hydrogen peroxide in methanol and blocked with a biotin-blocking kit (DAKO, Germany), incubated overnight with MTR4 polyclonal antibody (1:200, ab70551; Abcam) in a moist chamber at 4 °C. After washing three times with PBS, the slides were incubated with biotinylated goat anti-mouse antibodies for 1 h. The slides were stained with DAKO liquid 3,3′-diaminobenzi-dine tetrahydrochloride (DAB), followed by their counterstaining with Mayer’shematoxylin and examined under a microscope. The protein level of MTR4 was determined by semi-quantitative IHC detection.

Cell cycle analysis. Cells were seeded onto 6-well plates at a density of 1×10^6 cells/well and cultured with FBS free RPMI-1640 media for 12 h. Cells were fixed with Cell Cycle Staining Kit (MultiSciences, China CCS012) and according to the manufacturer’s instructions. Cell cycle analysis was performed using a flow cytometry (FACScan, BD Biosciences), and analyzed with the

ModFIt LTV4.1.7 software (BD Biosciences). Cell debris and dead cells were gated out for cell cycle analysis.

RNA-seq analysis. We mapped RNA-seq reads to the human genome (H. sapiens, GRCh38) and transcriptome (Ensembl, release 84) using the software hisat2 (v2.1.0) with the default parameters.

Quantification and statistic analysis. The statistical significance of Kaplan–Meier survival plot was determined by log-rank analysis. Univariate and multivariate analyses were based on the Cox proportional hazards regression model. Statistical significance in tumor growth rates was tested by repeated measure ANOVA. To compare the tumor weights between two groups, non-parametric Mann–Whitney test was used. For other experiments, significant difference was determined by t-test and ANOVA. Asterisks indicate significant difference: ns, not significant. We assumed normal distribution for the data having a small number. All statistical analyses of patient’s survival were performed with IBM SPSS Statistics 20.0 (IBM). Other analyses were performed with PRISM. N was indicated in the Fig.ure legends. No statistic method was used for determining sample size, blinding and randomizing.

Biological materials availability. All biological materials can be obtained by sending the request to the corresponding authors.
